# Gab2 plays a carcinogenic role in ovarian cancer by regulating CrkII

**DOI:** 10.1186/s13048-023-01152-y

**Published:** 2023-04-21

**Authors:** Yi Yin, Li Zhang, Yong Li, Can Zhang, Aiqin He

**Affiliations:** 1 Department of Gynecological Oncology, The Affiliated Tumor Hospital of Nantong University, Nantong Tumor Hospital, Nantong, Jiangsu China; 2 Department of Cancer Research Center, The Affiliated Tumor Hospital of Nantong University, Nantong Tumor Hospital, Nantong, Jiangsu China

**Keywords:** Gab2, CrkII, Ovarian cancer

## Abstract

**Objective:**

To detect the expression of Growth factor binding protein 2 associated binding protein 2 (Gab2) and CT10 regulator of kinase II (CrkII) in ovarian cancer and analyze their clinical significance. To explore the effects of Gab2 and CrkII on the biological behavior of ovarian cancer cells. To analyze the possible molecular mechanism of Gab2 in the development of ovarian cancer.

**Methods:**

Immunohistochemistry was used to detect the expression of Gab2 and CrkII in ovarian cancer. Chi square test was used to analyze the correlation between Gab2, CrkII and clinical parameters. Using Cox regression model to evaluate the risk factors affecting the prognosis. To analyze the correlation between Gab2, CrkII and survival rate by Kaplan–Meier. Cell experiments were preformed to explore the effects of Gab2 and CrkII on the biological behavior of cells. The interaction between Gab2 and CrkII was explored by immunoprecipitation.

**Results:**

Immunohistochemistry revealed that high expression of Gab2 and CrkII in ovarian cancer. Patients with high expression of Gab2 or CrkII had higher International Federation of Gynecology and Obstetrics (FIGO) stage, grade and platinum-resistance recurrence. Multivariate analysis showed that Gab2 and CrkII were independent prognostic factors. Kaplan–Meier curve showed that the higher Gab2 and CrkII were, the poor prognosis the patients had. We observed that the overexpression of Gab2 and CrkII promoted the proliferation, metastasis and reduced chemosensitivity of cells. Conversely, the knockdown of Gab2 and CrkII resulted in the opposite results. In CrkII-knockdown cells, we found that Gab2 mediates biological behavior through CrkII.

**Conclusions:**

The expression of Gab2 and CrkII increase in ovarian cancer. The higher expression of Gab2 and CrkII predict the poor prognosis of patients. Gab2 and CrkII promote the proliferation and migration and reduce the chemosensitivity of cells. Gab2 regulates the biological behaviors of ovarian cancer cells through CrkII.

## Highlights

The higher expression of Gab2 and CrkII predict the poor prognosis of patients with ovarian cancer.

Gab2 and CrkII promote the proliferation and migration and reduce the chemosensitivity of cells.

Gab2 may be a potential target for the treatment of ovarian cancer.

## Introduction

Ovarian cancer is one of the most common malignant tumors of female reproductive system. In 2020, there were more than 300,000 new cases of ovarian cancer worldwide and more than 200,000 deaths worldwide [1]. Most patients are at an advanced stage or have distant metastasis when they are diagnosed. This reduces the survival time of patients. The standardized treatment for ovarian cancer is the debulking surgery and the chemotherapy based on platinum after operation. Although ovarian cancer patients achieve a complete response after treatment, most patients develop chemotherapy resistance over time, resulting in multiple relapses and even death. Therefore, it is of great significance to find new potential targets for the treatment of ovarian cancer.

Gab family includes Gab1, Gab2 and Gab3 in mammals [2]. Gab2, scaffold protein, consists of PH domain, the proline-rich domain and multiple tyrosine residues. Several studies have shown that Gab2 plays a key role in cellular signaling pathways [3], including erythropoietin, thrombopoietin and et al. [4]. As reported, the expression of Gab2 was up-regulated in many malignant tumors, such as breast cancer [5], ovarian cancer [6], liver cancer [7] and melanoma [8]. In addition, studies had shown that Gab2 was expressed in a stage, grade and histological type dependent manner in ovarian cancer and was related to progression free survival in patients [9]. In the previous study, we found that Gab2 was increased in ovarian cancer tissues. Although the role of Gab2 in ovarian cancer has been rarely reported, more studies are needed to explore the possible molecular mechanism of Gab2 mediating malignant behavior of tumor cells.

The common structural feature of Crk family is that it is composed of SH2 and SH3 domains [10]. CrkII protein is composed of an SH2 domain and two SH3 domains. CrkII not only participates in the development of heart and skull, plays an important role in embryonic development [11], but also participates in the adhesion, proliferation and migration of tumor cells [12]. The study reports that CrkII can activate Rac1 and then promote the malignant biological behaviors of ovarian cancer cells [13]. However, there are few studies on the prognostic value of CrkII in patients with ovarian cancer.

In this study, the expression of Gab2 and CrkII in ovarian cancer tissue chip were detected by immunohistochemistry for the first time. And the relationship between Gab2, CrkII and clinical parameters and prognosis of patients were analyzed. Then, cell experiments were performed to further explored the effects of Gab2 and CrkII on biological behaviors of cells. Then, we analyzed the possible molecular mechanism of Gab2 on the biological behavior of ovarian cancer cells.

## Methods

### Patients

The ovarian cancer tissues and adjacent tissues (normal fallopian tube tissues not invaded determined by pathology) of 119 patients with ovarian cancer who underwent surgery in Affiliated Tumor Hospital of Nantong University from January 2013 to January 2016 were collected. Inclusion criterias: ⑴Epithelial ovarian cancer was diagnosed by pathology. ⑵The clinical data and follow-up data were completed. Exclusion criterias: ⑴Combined with or secondary to other malignant tumors. ⑵The clinical data and follow-up data were deficient.

Platinum sensitive recurrence was defined as the recurrence occurring more than 6 months after drug withdrawal, while platinum resistant recurrence was defined as the recurrence occurring within 6 months after drug withdrawal. Overall survival (OS) was defined as the time from the beginning of treatment to the end of death or follow-up. Progression free survival (PFS) was defined as the time between the beginning of treatment and the observation of disease progression or death from any cause. The deadline for follow-up was December, 2020.

### Immunohistochemical staining

The chips were incubated at 85 °C for 1 h. All chips were dewaxed and dehydrated. The alkaline repair solution was heated for antigen repair for 3 min. The chips were cooled to room temperature and blocked by peroxidase blocker at room temperature for 20 min, and incubated by the primary antibodies (dilution concentration was 1:50), Gab2 (Abcam, ab235932) and CrkII (Abcam, ab45136) at 4℃ overnight. The next day, the chips were rewarmed to room temperature, incubated by the secondary antibody at room temperature for 20 min, and flushed with PBS buffer 3 times for 5 min each time. All chips were colorated by DAB for 2 min, and stained by hematoxylin for 30 s. All chips were dehydrated, debenzene removed and sealed. All chips were re-read by 2 senior pathologists.

The positive standard of staining was cytoplasmic staining, and the negative standard was no cytoplasmic staining, regardless of whether the cell membrane and nucleus were stained or not. According to staining intensity, they were divided into non staining, light yellow, yellow and brown. The scores were 0, 1, 2 and 3. The percentage of positive cells were < 20%, 21% ~ 50%, 51% ~ 75% and > 75%, and the scores were 1, 2, 3 and 4. The comprehensive score was the product of the two indicators. Based on the comprehensive score, the optimal cut-off value was calculated according to receiver operating characteristic curve (ROC) and Youden index. According to the optimal cut-off value (cut off = 4), they were divided into high expression group (≥ 4) and low expression group (< 4) of Gab2, high expression group (≥ 4) and low expression group (< 4) of CrkII.

### Cell culture and transfection

Ovarian cancer cells (SKOV3, A2780 and HO8910) and ovarian surface epithelial cells (IOSE80), were obtained from National Collection of Authenticated Cell Cultures. SKOV3 and A2780, HO8910 and IOSE80 cell lines were cultured in DMEM medium, RPMI-1640 medium, supplement with 10% FBS, 100 μg/ml streptomycin and 100 μg/ml penicillin in 5% CO_2_ at 37 °C. Gab2 overexpression virus and CrkII knockdown virus were synthesized by GENE (Shanghai, China). Gab2 siRNA and CrkII overexpression plasmid were synthesized by OBiO (Shanghai, China). The vector was transfected into ovarian cancer cells A2780 and SKOV3 with Lipofectamine™ 2000 (Thermo Fisher Scientific).

### Western Blot

The lysate was added to the cells for 5 min. The supernatant was absorbed and stored at -20℃. The concentration of the extracted protein samples was determined by BCA method. SDS-PAGE electrophoresis device and configurate gel were assembled for electrophoresis. Protein sample was incubated in boiling water bath for 5 min. The sample loading amount in each hole was 15 μl protein, electrophoresis at constant pressure of 80 V for 1.5 h. The PVDF membrane was cut to a proper size, soaked with methanol, and then placed in ddH_2_O, soak in buffer for 10 min. The film was turned at 300A for 90 min. Hold PVDF membrane was placed in skimmed milk powder solution and incubated in a shaking table at room temperature for 2 h; Then the PVDF membrane was placed in the primary antibody and combined at 4℃ for overnight; PVDF membrane was placed in secondary antibody and combined at room temperature for 2 h. Color rendering. The primary antibodies of Gab2 and CrkII were diluted at 1:1000,GAPDH (Proteintech, 60004-1-Ig) was diluted at 1:2000 and the secondary antibodies, Mouse (Absin, abs20039) and Rabbit (Absin, abs20040), were diluted at 1:2000.

The relative band intensity of Gab2 in ovarian cancer cells A2780, SKOV3 and HO8910 were calculated by using image J software with reference to the band intensity of Gab2 expression in IOSE80. Similarly, the relative band intensity of CrkII in ovarian cancer cells A2780, SKOV3 and HO8910 were calculated. One-way ANOVA was used in the comparison.

### Cell proliferation assay

Cells (5 × 10^3^) were cultured into 96-well plates and incubated for 24 h, 48 h or 72 h. Then, CCK-8 solution was added into 96-well plates. After 2 h, the OD in each well was determined at 450 nm.

### Migration assay

1 × 10^5^ cells were seeded into the upper chamber of a transwell chamber in basal medium. The lower chamber was supplemented with medium containing 10% FBS. The cells were cultured for 72 h, and the chamber was fixed with 4% paraformaldehyde for 30 min. The cells were stained with 1% crystal violet for 15 min. Then the number of invaded cells was counted under a microscope.

### Cell viability assay

Cells (5 × 10^3^) were cultured into 96-well plates. After the cell adheres to the wall, the cells were treated with different concentrations of carboplatin (100, 300, 500, 700, 900, 1200 ug/ml) and cultured for 48 h. Then, CCK-8 solution was added into 96-well plates. After 2 h, the OD in each well was determined at 450 nm.

### Immunoprecipitation

The lysate was added to the cells for 30 min. The cell was collected and centrifuged at 12,000 rpm/30 min. The supernatant was taken after centrifugation. A small amount of lysate was taken for Western Blot. The remaining lysate was divided into two parts. 1 μl of corresponding antibody and 10–50 μl of protein A/G were added to the remaining lysate, and shake slowly for 4℃ overnight. After immunoprecipitation reaction, centrifugation was carried out at 3000 rpm/5 min at 4℃, and protein A/G was centrifuged to the bottom of the tube, suck up the supernatant. Protein A/G was washed 3–4 times with 1 ml lysate; Finally, 15 μl of 2 × SDS sampling buffer were added and cooker for 10 min. Western Blot analysis was performed.

### Statistical analysis

Statistical analysis were performed using SPSS 26.0. McNemar’s test was used to analyze the expression differences of Gab2 and CrkII in ovarian cancer tissues and adjacent tissues. Spearman method was used to analyze the correlation between Gab2 and CrkII expression. The associations between Gab2 and CrkII and clinical parameters were measured by Chi-square test. Cox regression model was used to analyze the risk factors affecting the prognosis of patients. Kaplan–Meier method and Log-rank test were used to draw the survival curve. T-test was used for comparison between the two groups. In vitro experiments were repeated at least three times and data were presented as mean ± SD. *P* < 0.05 was considered statistically significant.

## Results

### Gab2 and CrkII overexpression in ovarian cancer tissues

**Fig. 1:**
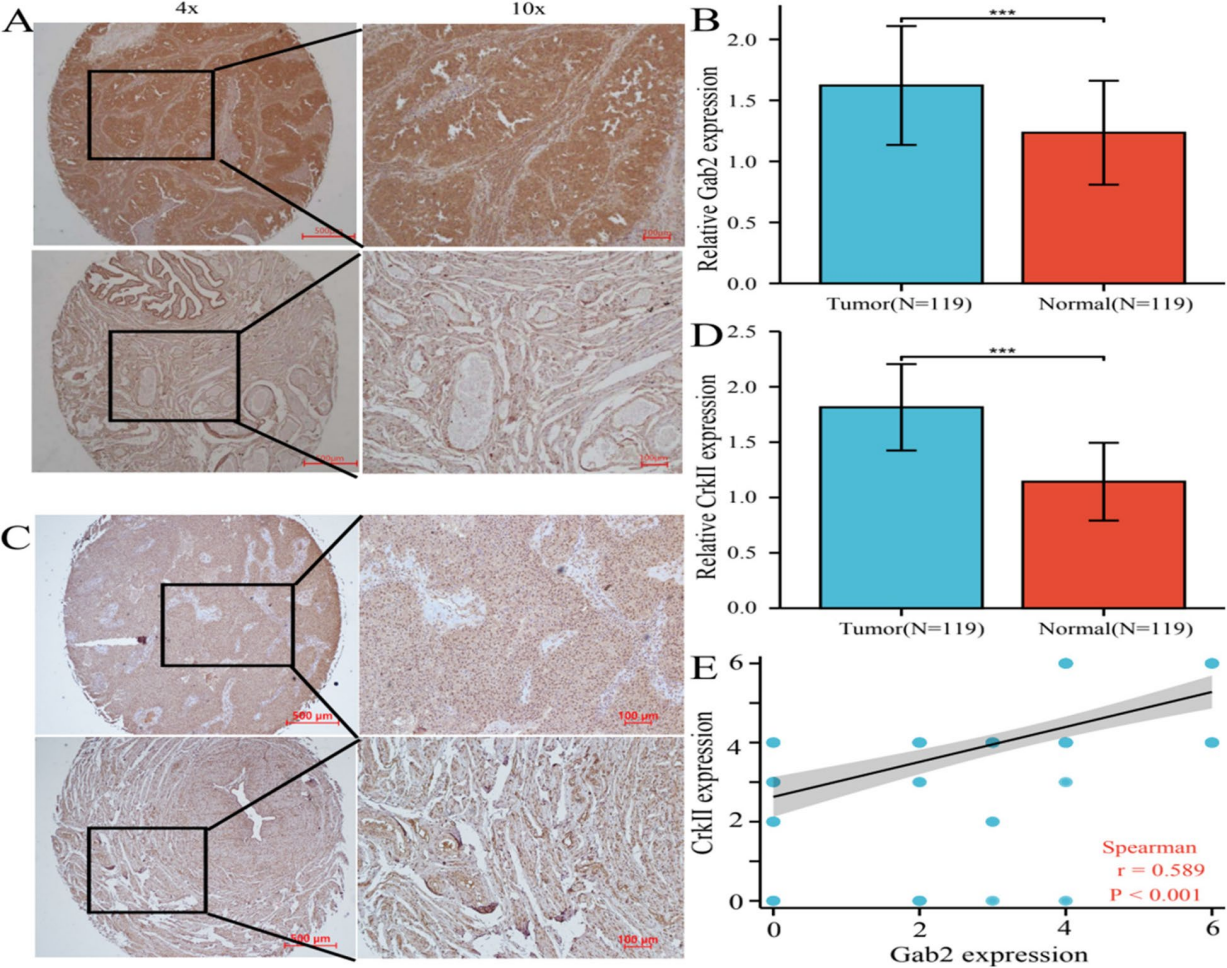
The expressions of Gab2 and CrkII in ovarian cancer were detected by immunohistochemistry. **A** Typical diagram of Gab2 immunohistochemical results (above: ovarian cancer tissue, below: adjacent tissues). **B** Expression of Gab2 in ovarian cancer tissue and adjacent tissues. **C** Typical diagram of CrkII immunohistochemical results (above: ovarian cancer tissue, below: adjacent tissues). **D** Expression of CrkII in ovarian cancer tissue and adjacent tissues. **E** The relationship between Gab2 and CrkII. The X-axis and Y-axis represent the comprehensive score of Gab2 and CrkII expression in ovarian cancer tissues, respectively

Immunohistochemical results showed that the expression of Gab2 was increased in 74 ovarian cancer tissues, while 28/119 cases had strong/moderate staining in adjacent tissues. The increased expression of CrkII was observed in 97 ovarian cancer tissues, while CrkII was increased in 17 adjacent tissues (Table 1). Results as shown in Fig. 1A-D, compared with adjacent tissues, the expression of Gab2 and CrkII were increased in ovarian cancer tissues (*P* < 0.001). In addition, we found that there was a positive correlation between the expression of Gab2 and CrkII in ovarian cancer(*r* = 0.589, *P* < 0.001) (Fig. 1E).

### Association between Gab2 and CrkII expression and clinical parameters

Chi square test was used to analyze the correlation between Gab2 and CrkII and clinical parameters. As showed in Table 1, Gab2 was correlated with FIGO stage *(P* = 0.023*)*, grade (*P* = 0.001), platinum resistance (*P* < 0.001), while CrkII was related to FIGO stage (*P* = 0.049), grade (*P* = 0.001), treatment (*P* = 0.04) and platinum resistance (*P* < 0.001).

**Table 1:** Relationship between Gab2, CrkII, and clinical characteristics of ovarian cancers

	N	Gab2 expression			CrkII expression		
		Low	High	*P* Value	Low	High	*P* Value
Age				0.404			0.694
< 57	55	23	32		11	44	
≥ 57	64	22	42		11	53	
Menopause				0.885			0.454
NO	23	9	14		3	20	
Yes	96	36	60		19	77	
FIGO stage				0.023			0.049
I-II	31	17	14		12	19	
III-IV	88	28	60		10	78	
Histological subtype				0.054			0.563
Serous	102	35	67		18	84	
Non-serous	17	10	7		4	13	
Grade				0.001			0.001
Low-grade	14	11	3		7	7	
High-grade	105	34	71		15	90	
Ascites				0.404			0.180
No	55	23	32		13	42	
Yes	64	22	42		9	55	
Treatment				0.697			0.040
PDS	42	17	25		12	30	
NACT + IDS	77	28	49		10	67	
Platinum resistance				< 0.001			< 0.001
NO	72	43	29		20	52	
Yes	47	2	45		2	45	
Resistance and progression				0.594			0.898
NO	10	3	7		2	8	
Yes	109	42	67		20	89	
lymph node metastasis				0.840			0.958
No	86	33	53		16	70	
Yes	33	12	21		6	27	

### Cox regression model was used to analyze the risk factors of OS in patients

As showed in Table 2, univariate analysis showed that FIGO stage (*P* < 0.001), grade (*P* = 0.038), platinum resistance (*P* < 0.001), Gab2 (*P* < 0.001) and CrkII (*P* < 0.001) expression could affect the OS of patients. Multivariate analysis showed that FIGO stage (*P* = 0.029), grade (*P* = 0.012), Gab2 (*P* = 0.007) and CrkII (*P* < 0.001) expression were independent prognostic factors in patients.

**Table 2:** Univariate and multivariate analyses of factors in predicting OS

	Univariate		Multivariate	
	HR (95% CI)	*P* Value	HR (95% CI)	*P* Value
Age				
< 57	Ref			
≥ 57	1.057(0.615–1.815)	0.842		
Menopause				
NO	Ref			
Yes	0.827(0.435–1.575)	0.564		
FIGO stage				
I-II	Ref		Ref	
III-IV	14.035(5.846–33.699)	< 0.001	3.267(1.127–9.518)	0.029
Histological subtype				
Serous	2.425(0.875–6.726)	0.089		
Non-serous	Ref			
Grade				
Low-grade	Ref		Ref	
High-grade	1.223(1.054–1.917)	0.038	2.469(0.913–5.999)	0.012
Ascites				
No	Ref			
Yes	1.559(0.894–2.717)	0.117		
Treatment				
PDS	Ref			
NACT + IDS	1.476(0.810–2.691)	0.203		
Platinum resistance				
NO	Ref			
Yes	5.449((3.140–9.455)	< 0.001		
Resistance				
NO	Ref			
Yes	0.713(0.348–1.462)	0.356		
lymph node metastasis				
No	Ref			
Yes	1.401(0.779–2.521)	0.260		
CrkII expression				
Low	Ref		Ref	
High	19.904(10.087–39.274)	< 0.001	15.763(6.913–35.943)	< 0.001
Gab2 expression				
Low	Ref		Ref	
High	10.320(5.730–18.589)	< 0.001	2.510(1.283–4.910)	0.007

### Gab2 and CrkII were associated with PFS and OS in patients

**Fig. 2:**
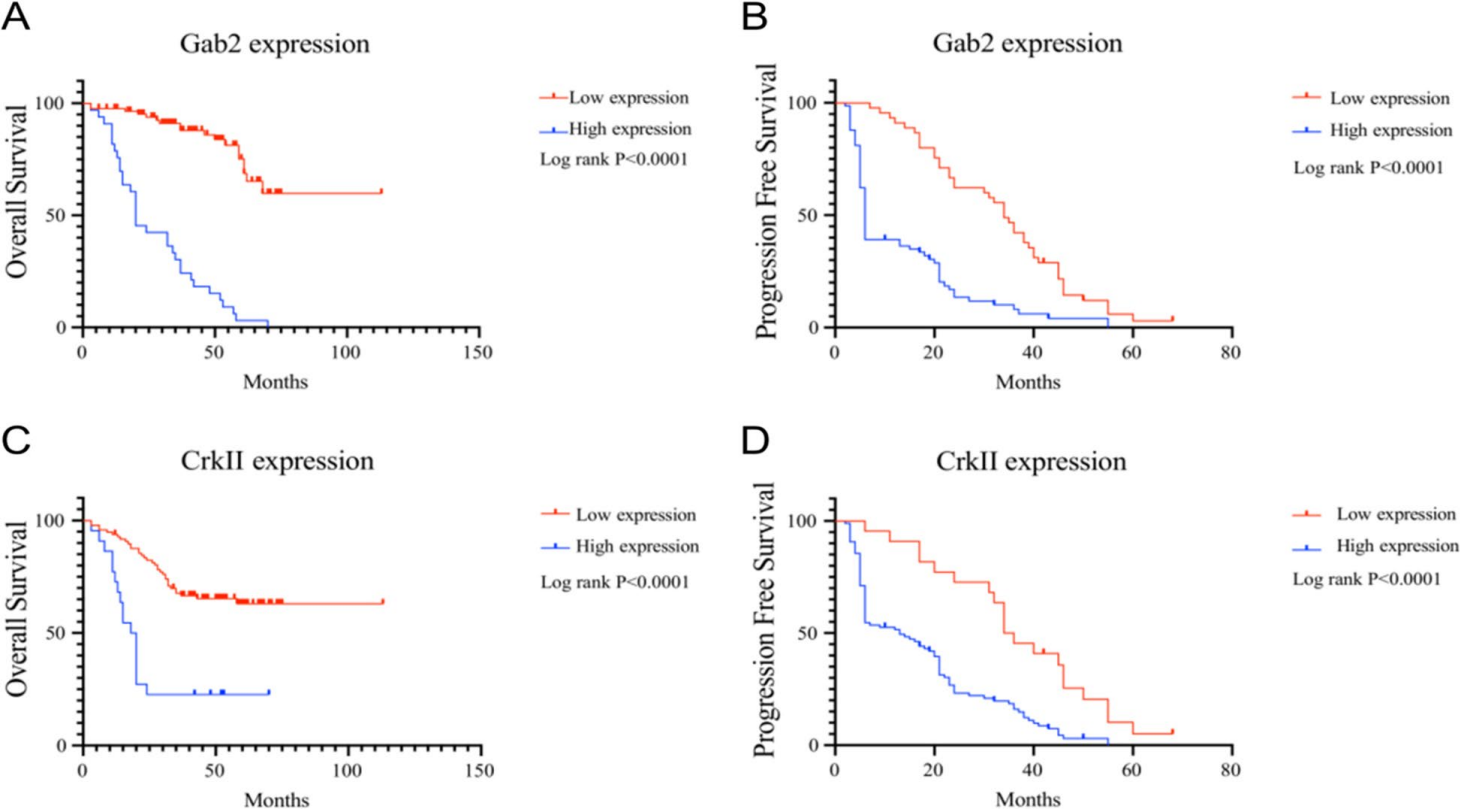
Relationship between Gab2 and CrkII and survival rate of patients with ovarian cancer. **A** Relationship between Gab2 and OS in patients. **B** Relationship between Gab2 and PFS in patients. **C** Relationship between CrkII and OS in patients. **D** Relationship between CrkII and PFS in patients

Kaplan–Meier analysis showed that both Gab2 and CrkII overexpression was associated with shorter OS and PFS (*P* < 0.05) as shown in Fig. 2A-D.

### Expression of Gab2 and CrkII in ovarian cancer cells

**Fig. 3:**
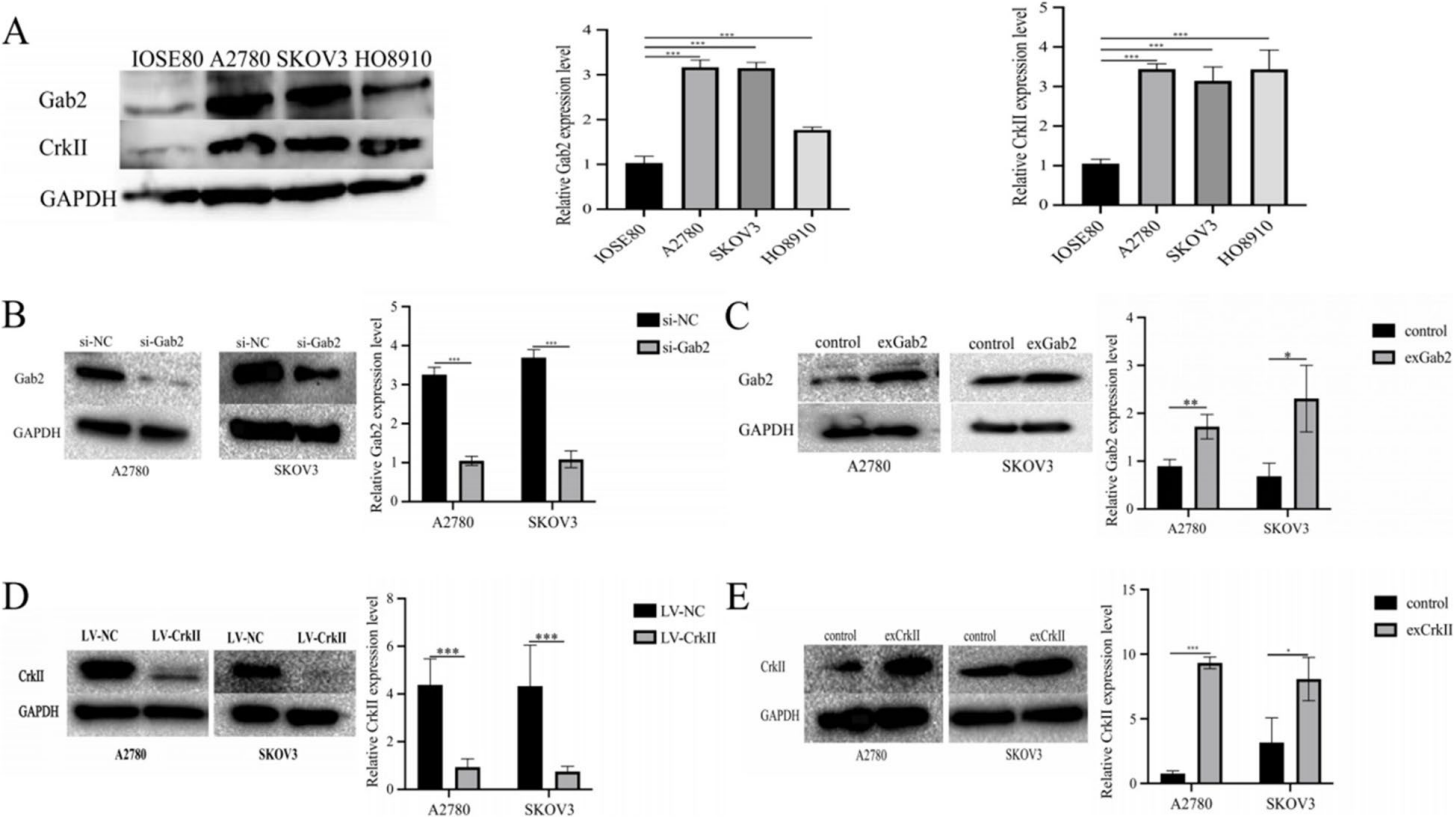
Expression of Gab2 and CrkII in ovarian cancer cell lines. **A** Western Blot was used to analysis of Gab2 and CrkII expression in ovarian cancer cell lines (A2780, SKOV3 and HO8910) and ovarian surface epithelial cells (IOSE80). **B** The expression of Gab2 in A2780 and SKOV3 transfected with Gab2 siRNA was elevated. **C** Evaluating the expression level of Gab2 in SKOV3 and A2780 cells were transfected with Gab2 overexpression virus. **D** The expression of CrkII in A2780 and SKOV3 transfected with CrkII knockdown virus was tested. **E** Testing the expression of CrkII in A2780 and SKOV3 were transfected with CrkII overexpression plastid

We next detected the expression of Gab2 and CrkII in cells. The results showed that compared with IOSE80, the expression of Gab2 and CrkII in A2780, SKOV3 and HO8910 were increased (*P* < 0.001) (Fig. 3A). We selected A2780 and SKOV3 for transfection. Then, the transfection efficiency was detected by Western Blot (Fig. 3B-E).

### Effect of Gab2 and CrkII on the biological behaviors of ovarian cancer cells

**Fig. 4:**
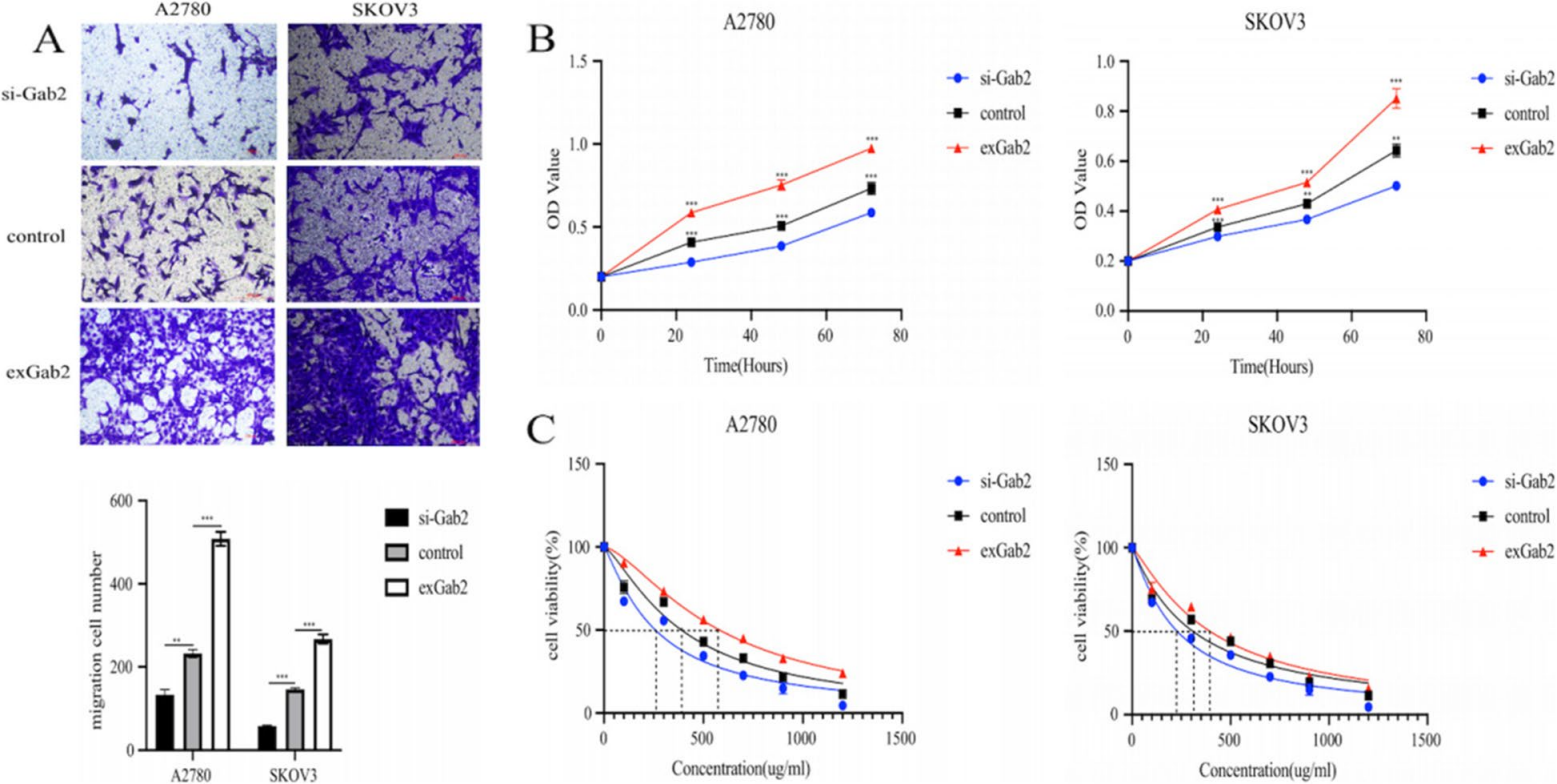
Effect of Gab2 on the biological behaviour of ovarian cancer cells. **A** Gab2 affects the migration ability of ovarian cancer cells. **B** Gab2 affects the proliferation of ovarian cancer cells. **C** Gab2 affects the sensitivity of ovarian cancer cells to carboplatin

To illuminate the effect of Gab2 on the biological behavior of ovarian cancer cells, we transfected siRNA or overexpressed virus on A2780 and SKOV3 cells. Then, the CCK8 and transwell experiments were performed. The results showed in Fig. 4A-B, downregulation of Gab2 can inhibit the migration (*P* < 0.001) and proliferation (*P* < 0.001). We added different concentrations of carboplatin to the transfected cells to detect cell activity. The results show that knockdown of Gab2 increased the chemosensitivity (IC50 = 389.7 vs 258.7 μg/ml, 307.6 vs 228.4 μg/ml). While overexpression of Gab2 can result in opposite results (Fig. 4C).

**Fig. 5:**
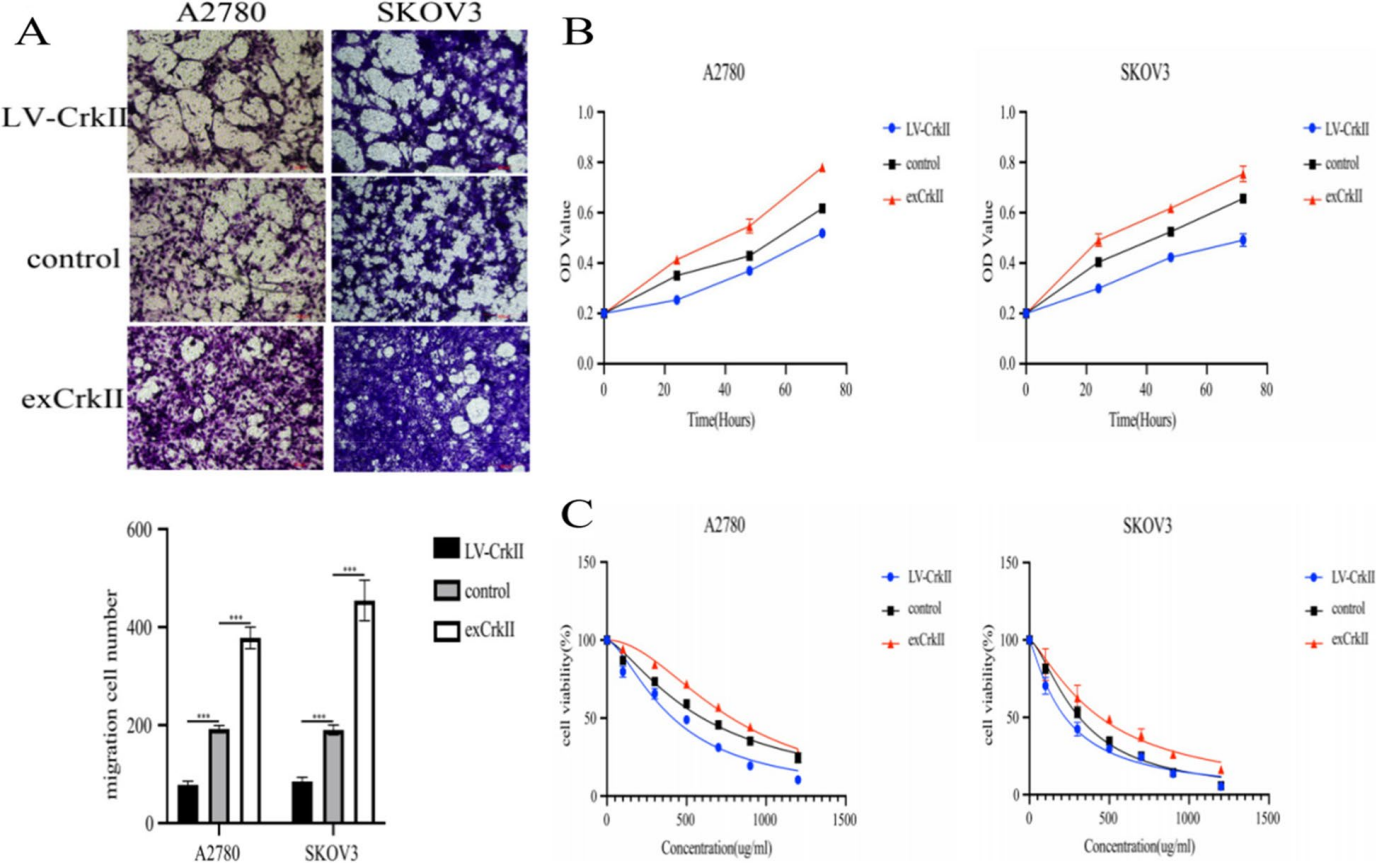
Effect of CrkII on the biological behaviour of ovarian cancer cells. **A** CrkII affects the migration ability of ovarian cancer cells. **B** CrkII affects the proliferation of ovarian cancer cells. **C** CrkII affects the sensitivity of ovarian cancer cells to carboplatin

Next, we explored the effect of CrkII on the biological behavior of ovarian cancer cells. The results are shown in the Fig. 5. The proliferation and migration ability of CrkII-knockdown cells decreased compared with the control (*P* < 0.05), while CrkII overexpression promoted cell proliferation and migration (*P* < 0.05) (Fig. 5A-B). In the cytotoxicity experiment, we found that the knockdown of CrkII increased the chemosensitivity (IC50 = 600.3 vs 414.7 μg/ml, 312.5 vs 223.8 μg/ml). On the contrary, the overexpression of CrkII decreased the chemosensitivity (IC50 = 600.3 vs 738.3 μg/ml, 312.5 vs 442.5 μg/ml) (Fig. 5C).

### Gab2 affects the biological function of cells through CrkII

**Fig. 6:**
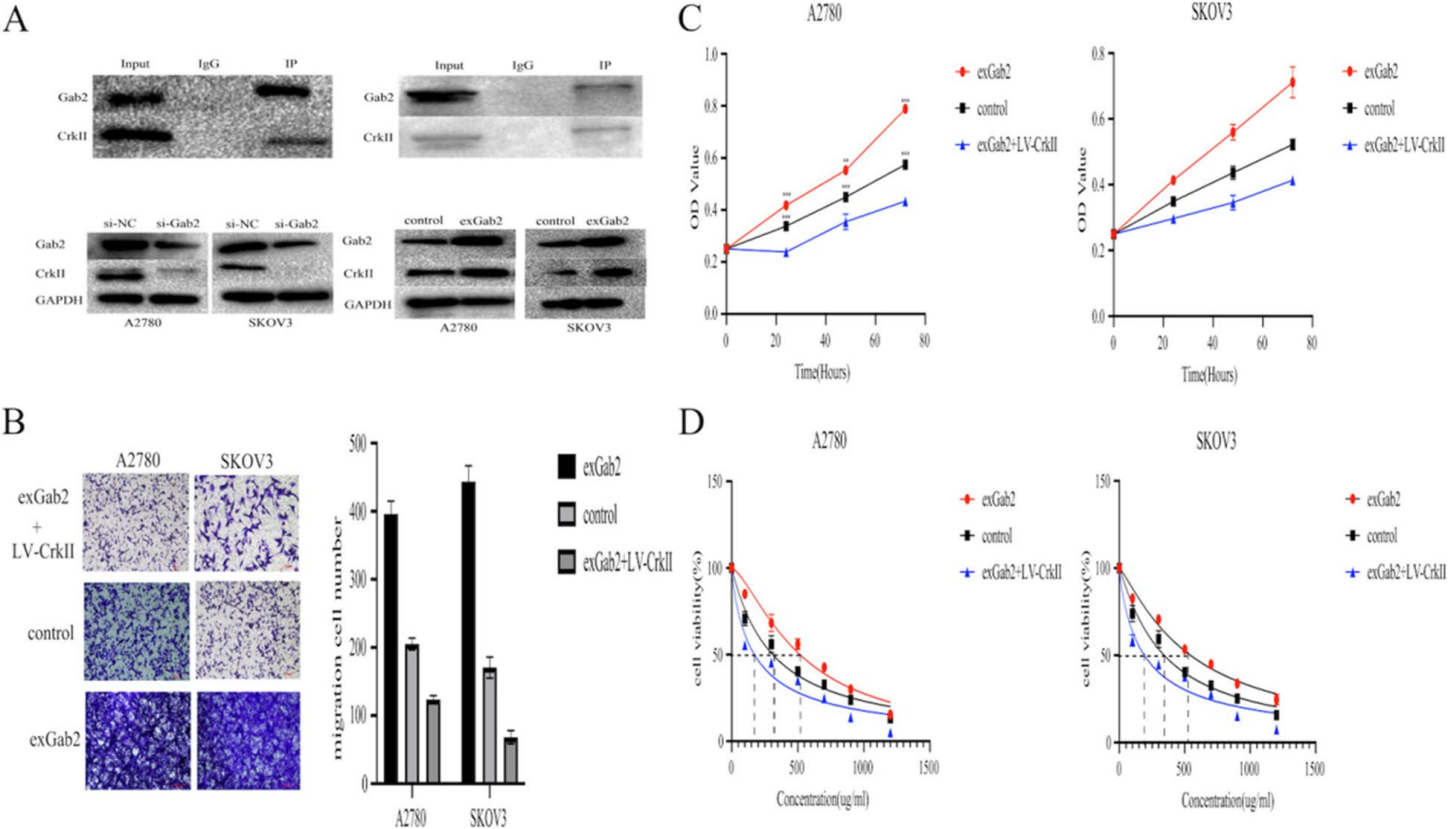
Gab2 affects the biological function of ovarian cancer cells through CrkII. **A** Co-IP was employed to determine the relationship between Gab2 and CrkII in A2780 and SKOV3. **B** Knockdown of CrkII reversed the migration of ovarian cancer cells induced by Gab2 overexpression. **C** Knockdown of CrkII reversed the proliferation of ovarian cancer cells induced by Gab2 overexpression. **D** The effect of Gab2 on the chemosensitivity of ovarian cancer cells depends on CrkII

To further explore the interaction between Gab2 and CrkII, we conducted immunocoprecipitation in ovarian cancer cells. As shown in Fig. 6A, we observed the interaction between Gab2 and CrkII. In addition, we also found that Gab2 could regulate the expression of CrkII. Then, Gab2 was overexpressed in CrkII-knockdown cells, we found that knockdown of CrkII reversed the proliferation and migration induced by Gab2 overexpression (Fig. 6B-C). We also observed that knockdown of CrkII prevented the inhibition of Gab2 overexpression on the chemosensitivity of cells (IC50 = 517.7 vs 311.5 vs 173 μg/ml, 543.8 vs 342.8 vs 191.4 μg/ml) (Fig. 6D).

## Discussion

At present, RAS/ERK and PI3K/AKT signaling pathways are considered to be the two main pathways of Gab2. In the RAS/ERK pathway mediated by Gab2-SHP2 complex, SHP2 is an important binding effector protein downstream of Gab2. Activated Gab2 regulates a variety of biological processes by activating SHP2, including cell adhesion, hematopoietic cell migration and breast acinar growth [4]. In the PI3K/AKT signal pathway, Gab2 acts on p85 subunit of PI3K and induces PI3K activation [14]. Activated PI3K leads to the production of PIP3, enhances the membrane recruitment of Gab2 and promotes the activation of PI3K [11], forming a positive regulation loop to amplify the PI3K/AKT signal pathway [15]. It is reported that the increased of Gab2 promotes the migration and invasion of esophageal cancer cells through SHP2/ERK pathway [16]. Chen [17] et al. confirmed that overexpression of Gab2 increased the resistance of hepatoma cells to doxorubicin. Berke [9] et al. also confirmed that the expression of Gab2 is related to the DFS of patients with ovarian cancer. Fang Z [18] et al. demonstrated that Gab2 promotes cancer stem cell characteristics and metastatic growth of ovarian cancer by down-regulating miR-200c. Our previous experimental results also showed that the expression of Gab2 was up-regulated in ovarian cancer. This is consistent with previous research reports.

It was found that under the stimulation of cytokines such as epidermal growth factor, fibroblast growth factor, hepatocyte growth factor and neurotrophic growth factor, proteins containing tyrosine residues were phosphorylated, such as paxillin, p130Cas and scaffold protein Gab2. These phosphorylated proteins can bind to the SH2 domain of CrkII [19], and then bind to the downstream proline-rich domain proteins through the SH3 domain in CrkII, such as C3G and Dock180, to transmit signals to small GTPases, such as Rac, Rap and Rho, so as to complete the signal transmission, and then regulate the movement and adhesion of cells. It is reported that CrkII is abnormal in lung cancer, glioblastoma, oral squamous cell carcinoma, pancreatic cancer and salivary gland tumor [20]. The expression of CrkII is up-regulated in oral squamous cell carcinoma, which is related to T stage, N stage and depth of invasion, and the high expression of CrkII predicts the poor prognosis of patients. Cell experiments also confirmed that the high expression of CrkII promoted the migration ability of oral squamous cell carcinoma cells [21]. Matthew [22] et al. found that PAK1 inhibited the expression of 120-catenin and E-cadherin by inducing CrkII serine phosphorylation, so as to promote the adhesion and migration of non-small cell lung cancer cells.

This study explored the expression of Gab2 and CrkII in ovarian cancer through tissue chips for the first time, and analyzed their clinical significance. However, the tissue chips used in the study contained only 119 cases. Because the detailed time of recurrence in some cases is not clear, this study only analyzed the correlation between Gab2 and CrkII and OS. This study only preliminarily confirmed the interaction between Gab2 and CrkII, but the specific mechanism of their interaction is not clear. Therefore, it is necessary to further study the mechanism of Gab2 and CrkII interaction and its role in tumorigenesis and development.

We confirmed that Gab2 and CrkII, as oncogenes, promote the proliferation and metastasis and reduce the chemosensitivity of cells. We also found that Gab2 can mediate the proliferation, metastasis and chemosensitivity of ovarian cancer by regulating the expression of CrkII. These results suggested that Gab2 may be an important molecule in the development of ovarian cancer and may be a potential target for clinical treatment of ovarian cancer.

## Data Availability

The datasets were analysed during this period can be provided by the corresponding author according to reasonable requirements.

## References

[1] Sung H, Ferlay J, Siegel RL (2021). Global Cancer Statistics 2020: GLOBOCAN Estimates of Incidence and Mortality Worldwide for 36 Cancers in 185 Countries. CA Cancer J Clin.

[2] Yamada K, Nishida K, Hibi M (2001). Comparative FISH mapping of Gab1 and Gab2 genes in human, mouse and rat. Cytogenet Cell Genet.

[3] Akter S, Rahman MA, Hasan MN (2022). Recent advances in ovarian cancer: therapeutic strategies, potential biomarkers, and technological improvements. Cells.

[4] Ding CB, Yu WN, Feng JH (2015). Structure and function of Gab2 and its role in cancer (Review). Mol Med Rep.

[5] Fleuren ED, O'Toole S, Millar EK (2010). Overexpression of the oncogenic signal transducer Gab2 occurs early in breast cancer development. Int J Cancer.

[6] Wang Y, Sheng Q, Spillman MA (2012). Gab2 regulates the migratory behaviors and E-cadherin expression via activation of the PI3K pathway in ovarian cancer cells. Oncogene.

[7] Cheng J, Zhong Y, Chen S (2017). Gab2 mediates hepatocellular carcinogenesis by integrating multiple signaling pathways. FASEB J.

[8] Horst B, Gruvberger-Saal SK, Hopkins BD (2009). Gab2-mediated signaling promotes melanoma metastasis. Am J Pathol.

[9] Berkel C, Cacan E (2021). GAB2 and GAB3 are expressed in a tumor stage-, grade- and histotype-dependent manner and are associated with shorter progression-free survival in ovarian cancer. J Cell Commun Signal.

[10] Park T (2021). Crk and CrkL as therapeutic targets for cancer treatment. Cells.

[11] Liu D (2014). The adaptor protein Crk in immune response. Immunol Cell Biol.

[12] Feller SM (2001). Crk family adaptors-signalling complex formation and biological roles. Oncogene.

[13] Wang H, Linghu H, Wang J (2010). The role of Crk/Dock180/Rac1 pathway in the malignant behavior of human ovarian cancer cell SKOV3. Tumour Biol.

[14] Maus M, Medgyesi D, Kövesdi D (2009). Grb2 associated binder 2 couples B-cell receptor to cell survival. Cell Signal.

[15] Zhang TT, Li H, Cheung SM (2009). Phosphoinositide 3-kinase-regulated adapters in lymphocyte activation. Immunol Rev.

[16] Zhang Y, Pan E, Zhao C (2021). Synergism of HPV and MNNG repress miR-218 promoting Het-1A cell malignant transformation by targeting GAB2. Toxicology.

[17] Chen Y, Liu Q, Wu M (2016). GAB2 promotes cell proliferation by activating the ERK signaling pathway in hepatocellular carcinoma. Tumour Biol.

[18] Fang Z, Li T, Chen W (2019). Gab2 promotes cancer stem cell like properties and metastatic growth of ovarian cancer via downregulation of miR-200c. Exp Cell Res.

[19] Bos JL, Rehmann H, Wittinghofer A (2007). GEFs and GAPs: critical elements in the control of small G proteins. Cell.

[20] Zhou Z, Sun X, Guo C (2019). CRKII overexpression promotes the in Vitro proliferation, migration and invasion potential of murine hepatocarcinoma Hca-P cells. Oncol Lett.

[21] Yamada S, Yanamoto S, Kawasaki G (2011). Overexpression of CRKII increases migration and invasive potential in oral squamous cell carcinoma. Cancer Lett.

[22] Miller CT, Chen G, Gharib TG (2003). Increased C-CRK proto-oncogene expression is associated with an aggressive phenotype in lung adenocarcinomas. Oncogene.

